# Outcome and Complications of Endoscopic Dacryocystorhinostomy without Stenting

**DOI:** 10.12669/pjms.295.3393

**Published:** 2013

**Authors:** Muhammad Ayoob, Khalid Mahida, Qurat ul-ain, Zafar Dawood

**Affiliations:** 1 Muhammad Ayoob, FCPS, Assistant Professor of Ophthalmology, Ziauddin University Hospital, Keamari, Karachi, Pakistan.; 2 Khalid Mahida, FCPS, Associate Professor of E.N.T Department, Ziauddin University Hospital, Keamari, Karachi, Pakistan.; 3 Dr.Qurat-ul-ain, FCPS, Assistant Professor of Ophthalmology, Ziauddin University Hospital, Keamari, Karachi, Pakistan.; 4 Zafar Dawood, FCPS, Associate Professor of Ophthalmology, Ziauddin University Hospital, Keamari, Karachi, Pakistan.

**Keywords:** Epiphora, Dacryocystitis, Nasolacrimal duct, Endoscopic, Endonasal, Dacryocystorhinostomy

## Abstract

***Objectives:*** To determine the outcome and complications of endoscopic dacryocystorhinostomy without stenting at Ziauddin University Hospital, Keamari, Karachi, Pakistan.

***Methodology:***Fifty Patients who underwent endoscopic dacryocystorhinostomy from August 2011 to July 2012 at Department of Ophthalmology, Ziauddin University Hospital, Keamari, Karachi were included.Data regarding the Outcomes and complications were collected and analyzed.

***Results:***Among the 50 patients there were 38%(n=19) males and 62%(n=31) females.Their age ranged from 15 to 60 years, mean age was 43.54 ± 9.36 years. Majority of patients were in the age ranging from 31 to 50 years. The success rate after 6 months of surgery was 92% without any significant complications.

***Conclusion:***Endoscopicdacryocystorhinostomy without stent is minimally invasive technique with less complications and good success state. In addition it gives no facial scar.

## INTRODUCTION

Nasolacrimal duct obstruction most commonly presents with ephiphora,^1^^,^^2^ other symptoms are discharge from the eyes and swelling over the sac area. Watering due to nasolacrimal duct obstruction causes much disturbance for the patient but is not a serious problem. It is generally unilateral. Symptoms persist if the condition is not treated and may predispose to chronic or acute dacryocystitis.^3^ Conservative treatment like massage over the sac area does not relieve the symptoms. Syringing and probing also does not help but sometimes causes temporary relief of symptoms in patients with incomplete blockage of nasolacrimal duct. Treatment of nasolacrimal duct obstruction is dacryocystorhinostomy.^1^^,^^4^^,^^5^

As cited by Tan NC etal 2009; Mortimore S etal 1999,Toti in 1904 first described the procedure of external dacryocystorhinostomy.^5^^,^^6^External dacryocystorhinostomy is a gold standard traditional surgical approach to treat nasolacrimal duct obstruction.^3^^,^^7^ Success rate of other techniques is measured and compared with this method.^3^ Most Ophthalmologists believes that external dacryocystorhinostomy provides highest success rate as compared to other techniques.^7^

As cited by various studies intranasal dacryocystorhinostomy was first described by Caldwell in1893.^5^^,^^6^^,^^8^^,^^9^ Intranasal technique remained limited at that time due to poor visibility of intranasal anatomy. This technique gained popularity after introduction of high resolution fiber-optic endoscopes and rigid endoscopes with different degrees of angulations.^10^As cited by Tan NC et al 2009; Mortimore S et al 1999,McDonogh and Meiring introduced endoscopic trans-nasal dacryocystorhinostomy in 1989.^5^^,^^6^

Various changes in surgical procedure of dacryocystorhinostomy have been introduced to acquire a good surgical success rate.^1^ Basic concept of various procedures is to create a fistula between lacrimal sac and nasal cavity for the drainage of tears.^6^^,^^8^

Endonasaldacryocystorhinostomy is a surgical technique in which a fistula is created from inside the nasal cavity.^8^ It can be performed surgically using drill or rounguer to remove the bone or by laser.^9^

This procedure is now regularly being done at Ziauddin University Hospital, Keamari. Karachi. Our aim was to see the outcome and complications of endoscopic DCR at our center.

## METHODS

This study was conducted at Department of Ophthalmology and E.N.T, Ziauddin University Hospital, Keamari, Karachi from August 2011 to July 2012. Endoscopic dacryocystorhinostomy without stent was performed for epiphora due to nasolacrimal duct obstruction.

A complete history, examination of lids and adnexa, regurgitation test and syringing and probing was done in every patient. Endoscopic examination of nasal cavities was done for any nasal pathology especially mucosal disease, hypertrophied middle turbinate, nasal polyp, deviated nasal septum and anatomical variations of lacrimal sac that may produce hindrance during endonasal surgery. Endoscopic examination of the nasal cavities was performed while doing probing with vitrectomy light which helped to find out anatomical variations of lacrimal sac. 

Systemic diseases especially hypertension and diabetes were evaluated. All patients were explained about the difference and outcomes of both conventional and endonasal dacryocystorhinostomy and a written consent from the patients was then taken for endoscopic dacryocystorhinostomy.

***Inclusion criteria: ***Epiphora due to nasolacrimal duct obstruction and chronic dacryocystitis.

***Exclusion criteria:***

Absent both upper and lower punctum.

Both upper and lower canalicular obstruction.

Lid laxity leading to displacement of punctum.

Previous lacrimal surgery.

Traumatic or congenital bony deformity.

Patients below 15 years of age.

***Surgical technique: ***All surgeries were performed by a team of same Ophthalmologist and Otorhinologist under general anesthesia.Nostril of the affected side was packed with 2% xylocaine with adrenaline solution 10 minutes before starting the surgery. Both upper and lower punctum were dilated and 20 gauge vitrectomy light was passed through the upper punctum into the lacrimal sac. Nasal packing was removed and nasal cavity was entered with 4mm 0^0 ^Hopkin Karl Storz rigid nasal endoscope attached to an endoscopic video camera (Stryker endoscopy system). Trans-illumination of the nasal mucosa produced by the vitrectomy light in the lacrimal sac was localized by movements of the light inside the lacrimal sac, exact location and size of the lacrimal sac was marked. Using phaco knife 3.2mm, two parallel incisions were made, one incision just anterior and second incision posterior to the trans-illumination area produced by the vitrectomy light. Nasal mucosa and periosteum between these two incisions was then elevated from the bone with the periosteum elevator and 1x1.5 cm of the nasal mucosa overlying lacrimal sac was removed with upbitting Balaesky forceps. Using the Kerrisonrounguer the bone forming the lacrimal crest was nibbled and removed. The opening was then enlarged using the same rounguer or bone drill. Bony ostium was enlarged to an extent of approximately 1x1.5cm, bone overlying the upper part of nasolacrimal duct was also removed. The vitrectomy light was then clearly seen inside the lacrimal sac. Elevating (tenting) the nasal mucosa with vitrectomy light, a vertical incision was made with phaco knife avoiding injury to nasal mucosa to minimize the hemorrhage. Vitrectomy light was then passed in the nasal cavity. Nasal mucosa and medial wall of the sac was removed up to the extent that the cut margins of the sac were approximated with the margins of the nasal mucosa around the margins of bony ostium. Bone and medial wall of the sac was removed inferiorly up to the upper part of nasolacrimal duct to prevent sump syndrome.^6^ Small gel foam patch was packed inside the lacrimal sac to keep the lacrimal sac mucosa approximated with the nasal mucosa, light nasal packing was applied. Nasal packing and gel foam inside the lacrimal sac kept the flaps anastomosis in position, nasal packing was removed after 24 hours.

Postoperatively systemic antibiotic and analgesic were given for 05 days. Antibiotic (Moxifloxacin) eye drops for 04 weeks, nasal decongestant spray for the initial period and saline drops inside the nasal cavity to prevent crust formation were given. Patients were followed up regularly at week 1, week 2, week 4, week 6, 3 months and 6 months. Postoperatively patients symptoms were evaluated and endoscopic examination of nasal cavity performed on each visit with Karl Storz flexible endoscope to evaluate the healing process, size, shape and patency of the neo-ostium. Jone’s dye test and irrigation of the lacrimal passage was performed while performing examination of nasal cavity with endoscope. Any clot or debris obstructing the ostium was also removed.

## RESULTS

Endoscopic endonasal DCR without intubation was performed in 50 patients under general anesthesia. Out of 50 patients, 19 were males and 31 females. Patients age ranged from 15 to 60 years. Mean age was 43.54 ±9.36 years. Most of the patients were in the age range of 31 to 50 years. Complete relief from epiphora was observed in 92%(n=46). 

No significant intraoperative complication was observed in any one of these cases. Post operatively eyelid edema in two patients and mild nasal bleeding in three patients was observed and managed conservatively. Eight percent (n=4) had procedure failure. In one patient cause of procedure failure was granuloma formation at the site of neo-ostium. Two patients had fibrosis at the site of neo-ostium and one patient had hypertrophied middle turbinate, this patient was pre-operatively diagnosed as a case of chronic allergic rhinitis. Failed endoscopic DCR patients underwent dacryocystography post-operatively which showed blockage of neo-ostium.

Three patients had repeat endoscopic surgery with silicone tube insertion and one patient with history of chronic allergic rhinitis was managed conservatively. Neo-ostium opening was cleared post operatively by removing clot and debris and advised instillation of normal saline drops post-operatively.

Average time taken in primary endoscopic DCR surgery was 35 minutes ranging from 30-50 minutes. With the increased surgical experience time duration for surgery decreased gradually.

## DISCUSSION

Endonasaldacryocystorhinostomy have some advantages compared to external dacryocystorhinostomy.There is no skin incision, minimum tissue injury which is limited to the fistula site, short hospital stay, rapid rehabilitation and patient’s preference.^2^^,^^8^^,^^9^^,^^11^^,^^12^Endonasal technique requires time to acquire expertise of using endoscope, i.e. steep learning curve and high equipment cost.^11^

Proper pre-operative examination of nasal cavity is important for patient selection for this procedure. Nasal septum deviation causing narrow nasal cavity at the neo-ostium, connective tissue disorder,sarcoidosis, chronic sinus disease, mucocele, previous external dacryocystorhinostomy or other nasal surgery are the pre-operative risk factors.^11^ Severe nasal deformity and scarring of nasal mucosa are the basic contraindication for endonasal dacryocystorhinostomy.^11^

In this study complete relief from epiphora was observed in 92% (n=46).Success rate of our study is comparable to others.^3^^,^^5^^,^^10^^,^^13^^,^^14^Success rate of endonasal endoscopic DCR in other studies are as follows, Zaman M and colleagues^3^ reported 95% success rate, Tan NC and colleagues^5^ reported 95% success rate, Kakar V and colleagues^10^ reported 90% success rate, Yung MW and colleagues^13^ achieved 93% success rate, Massegur H and colleagues^14^ reported 92.7% success rate.

Only eight percent (n=4) had procedure failure, one patient had granuloma formation at the site of neo-ostium, two patients had fibrosis and one patient had hypertrophied middle turbinate. Similarly Ressionitisetal^11^ found obstruction of neo-ostium by granulation tissue or fibrosis as the most common cause of failure. Adhesion may also form between the flaps of nasal mucosa, flaps of lacrimal sac and sometimes between the nasal mucosa at the margins of ostium and nasal septum if there is damage to the nasal mucosa covering the nasal septum.^11^

No intra operative complications were observed in our study. In the literature, bleeding from the nasal cavity occurs if there is extensive damage to the lacrimal sac mucosa or mucosa of the nasal septum^7^^.^^10^, Orbital injury, especially when too much of the soft tissue is removed while removing the medial wall of the lacrimal sac^7^, recurrent infection if the bone covering the lower part of lacrimal sac is not removed completely^7^are the complications of endonasal DCR.

Post-operative outcomes like relief of the symptoms of epiphora, patency of ostium opening into the lacrimal sac positive Jone’s dye test are indicators of successful surgery.^6^^,^^11^By and large endonasal DCR without stent is an effective and safe method to treat nasolacrimal duct obstruction.

## CONCLUSIONS

Endonasal DCR without stent is considered as effective, safe and minimally invasive primary procedure for the treatment of nasolacrimal duct obstruction. This procedure has fewer complications and is well tolerated by the patient. In this procedure there is minimum damage to anatomical structures. Mutual efforts by Ophthalmologists and Otorhinologist made endonasal DCR a good alternative to external DCR with high success rate and comparable outcomes. Regular follow up are required to evaluate the process of wound healing and early detection of complications leading to failure of the procedure.

## References

[1] Angela MD (2010). Non laser endoscopic endonasaldacryocystorhinostomy with adjunctive mitomycin in nasolacrimal duct obstruction in adults. Ophthalmology.

[2] Muscatello L, Giudicem M, Spriano G, Tondini L (2009). Endoscopic Dacryocystorhinostomy:Personal experience. ActaOtorhinolaryngol.

[3] Zaman M, Babar TF, Abdullah A (2005). Prospective randomized comparison of dacryocystorhinostomy with and without intubation. Pak J Med Res.

[4] Simon GJB, Joseph J, Lee S (2005). External versus endoscopic dacryocystorhinostomy for acquired nasolacrimal duct obstruction in a tertiary referral center. Ophthalmology.

[5] Tan NC, Rajapaksa SP, Gaynor J, Nair SB (2009). Mechanical endonasaldacryocystorhinostomy - a reproducible technique. Rhinology.

[6] Mortimore S, Banhegy GY, Lancaster JL, Karkanevatos A (1999). Endoscopicdacryocystorhinostomy without siliconestenting. JR Coll Surg Edinb.

[7] Jin HR, Yeon JY, Choi MY ( 2006). Endoscopic dacryocystorhinostomy: Creation of a large marsupialized lacrimal sac. J. Korean Med Sci.

[8] Aslam S, Awan AH, Tayab M (2010). Endoscopic dacryocystorhinostomy: A Pakistani experience. Pak J Ophthalmol.

[9] Yuen KSC, Lam LYM, Tse MWY (2004). Modified endoscopic daryoystorhinostomy with posterior lacrimal flap for nasolacrimal duct obstruction.Hong Kong Med J. Hong Kong Med J.

[10] Kakar V, Chugh JP, Sachdeva S, Sharma N, Ramesh (2009). Endoscopic dacryocystorhinostomy with and without silicone stent: A comparative study. Internet J Otorhinolaryngol.

[11] Ressionitis T, Voros GM, Vasilios TK (2005). Clinical outcome of endonasal KTP laser assisted dacryocystorhinostomy. BMC Ophthalmol.

[12] Karim R, Ghabrial R, Lync TF, Tang B (2011). A comparison of external and endoscopic endonasaldacryocystorhinostomy for acquired nasolacrimal duct obstruction. Clin Ophthalmol.

[13] Yung MW, Hardman-Lea S (2002). Analysis of the results of surgical endoscopic dacryocystorhinostomy:effect of the level of obstruction. Br J Ophthalmol.

[14] Massegur H, Trias E, Adema JM (2004). Endoscopic dacryocystorhinostomy: modified technique. Otolaryngol Head Neck Surg.

